# Inhibitory Effect of Various Breads on DMH-Induced Aberrant Crypt Foci and Colorectal Tumours in Rats

**DOI:** 10.1155/2015/829096

**Published:** 2015-05-14

**Authors:** Guangying Qi, Sien Zeng, Tiri Takashima, Koichiro Nozoe, Megumi Shobayashi, Koji Kakugawa, Kaori Murakami, Hiroshi Jikihara, Lihua Zhou, Fumio Shimamoto

**Affiliations:** ^1^Department of Pathology (Affiliated Hospital), Physiopathology and Academic Administration, Guilin Medical University, Guilin, Guangxi 541004, China; ^2^Department of Health Sciences, Prefectural University of Hiroshima, Minami-ku, Hiroshima 734-8558, Japan; ^3^Research and Development of Baking, Andersen Institute of Bread & Life Co., Ltd., Hiroshima 739-0323, Japan; ^4^Department of Food Sciences and Biotechnology, Hiroshima Institute of Technology, Hiroshima 731-5193, Japan

## Abstract

Bread is rich in dietary fibre and many phytochemical compounds, which may influence chemoprevention of colon cancer. In the present study, we evaluated the effect of three kinds of bread on DMH-induced colorectal tumours in F344 rats. F344 rats were divided into four groups (Steinmetz Three-Grain bread, Steinmetz Country bread, White bread, and MF). All groups were injected with 1,2-dimethylhydrazine (DMH, 20 mg/kg body weight) once a week for 8 consecutive weeks from 5 weeks of age. To investigate the antioxidant effect of bread, the 2,2-diphenyl-1-picrylhydrazyl (DPPH) free radical scavenging rate of bread and the serum levels of 8-hydroxy-deoxyguanosine (8-OHdG) in rats were examined. The number of colorectal aberrant crypt foci (ACF) and the incidence of colorectal tumours were studied after 34 weeks of DMH treatment. The Steinmetz Three-Grain and Steinmetz Country bread groups had higher scavenging rates of the DPPH free radical and lower serum levels of 8-OHdG and incidence of ACF, adenomas, and adenocarcinomas of colon than the White bread and MF group. Steinmetz Three-Grain bread and Steinmetz Country bread have various ingredient combinations that may inhibit colorectal cancer progression.

## 1. Introduction

Among cancer diseases, colorectal carcinomas are the second and third most common causes of death of men and women, respectively, in developed countries [1]. Lifestyle and dietary patterns influence colon cancer risk both positively and negatively. Among the dietary factors, several plant-derived compounds have been found to provide colon cancer protection [2]; however, a high intake of animal fat increases the risk of colon cancer [3]. The promotion of a healthy lifestyle is one of the major goals of governments and international agencies worldwide. It has been suggested that diets high in fibre may reduce the risk of incident colorectal cancer (CRC) [4]. Other studies have shown that diets very low in fibre may increase the risk of incident CRC [5, 6]. Bread as a staple food product represents an important source of dietary fibre and various ingredients, which may influence chemoprevention of colon cancer. The potential of protection by fibre from foods in populations with current low intake may therefore be even greater than our findings, which predict a 40% reduction in CRC risk when fibre intake is approximately doubled [7]. Wholegrain cereals are rich in nutrients and many phytochemical compounds, with recognised health benefits, including dietary fibre, a number of phenolic compounds, lignans, vitamins and minerals, and other bioactive components [8]. However, it remains unclear which components of the diet are the most important, and it is necessary to determine the protective factors.

Steinmetz flour was developed by the German Steinmetz company 115 years ago. Grain only removes the hull; the aleurone layer, germ, and endosperm are preserved by the patented Steinmetz process. Scientific surveys have confirmed that residues of environmental pollution such as lead, bacteria, and bitter substances are removed by the Steinmetz process, while all vitamins and minerals are preserved (Steinmetz; http://www.premium-flour.com). In this study, we also tested nutritional content of various flours and found that Steinmetz flour contains a lot of vitamins and minerals. Steinmetz flour may have a health benefit and Steinmetz flour food may reduce the risk of incident colorectal cancer.

1,2-Dimethylhydrazine- (DMH-) induced colon carcinogenesis in rats is a widely used experimental model among cancer chemoprevention studies [9]. DMH-induced colon cancer is a multistep process involving a series of pathological alterations, such as formation of aberrant cryptic foci [10].

In the present study, we have evaluated the effect of two kinds of Steinmetz bread and White bread on DMH-induced colorectal tumours in F344 rats.

## 2. Materials and Methods

### 2.1. Animals

Forty-two 4-week-old male F344 rats were purchased from Charles River Japan Inc. (Kanagawa, Japan). The animals were cared for in compliance with the principles and guidelines of Ethical Committee for Animal Care and institutional animal ethical committee, in accordance with the Japan National Law on Animal Care and Use. Rats were housed in wire cages in a standard air-conditioned room with a 12-h light/dark cycle at 23 ± 2°C and 50 ± 10% humidity, with free access to food and drinking water. Body weight was recorded every 4 weeks. DMH was purchased from Tokyo Chemical Industry (Tokyo, Japan).

### 2.2. Diet

**Figure 1:**
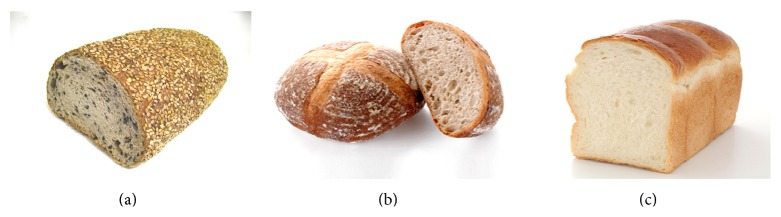
Bread photos. (a) Steinmetz Three-Grain bread. (b) Steinmetz Country bread. (c) White bread.

Three kinds of bread (Steinmetz Three-Grain bread, Steinmetz Country bread, and White bread; Andersen Institute of Bread & Life Co., Ltd., Hiroshima, Japan) and a basal diet (MF) (Oriental Yeast Co., Ltd., Tokyo, Japan) were used as the diet (Figure 1). The nutritional information and the combination of the raw materials of the breads are shown in Tables 1 and 2, respectively. The content of energy of 100 g Steinmetz Three-Grain bread, Steinmetz Country bread, and White bread is 256 kcal, 241 kcal, and 244 kcal, and dietary fiber is 8.2 g, 7.6 g, and 2.7 g (Table 1). White bread was made from white wheat flour (100%) only; Steinmetz Country bread was made from Steinmetz flour (72.6%) and rye flour (27.4%); and Steinmetz Three-Grain bread was made from Steinmetz flour (75%), whole-wheat flour (15%), and rye flour (10%) and also sesame was added (Table 2(a)). In this study, we also tested nutritional content of each ingredient of the breads and found that Steinmetz flour, rye flour, and whole-wheat flour and sesame contain a lot of vitamins and minerals; however, white wheat flour contains small quantities of vitamins and minerals.

### 2.3. Experimental Protocol

The 42 male rats were randomly divided into four groups (Table 3) and fed a bread diet (Group A: Steinmetz Three-Grain bread diet; Group B: Steinmetz Country bread diet; Group C: White bread diet; Group D: MF diet) (Figure 1, Table 3). There was no difference in mean body of four group rats. The first week before the start of the experiment, all rats were fed a basal diet (MF). The second week before the start of the experiment, the rats were fed either a basal diet (MF) or various breads diet. DMH was dissolved in 0.9% NaCl solution and the pH was adjusted to 6.5 with NaHCO_3_. All the experimental animals were injected with DMH (20 mg/kg body weight) once a week for 8 consecutive weeks from 5 weeks of age. All the rats were sacrificed 32 weeks after commencement of DMH administration.

### 2.4. 2,2-Diphenyl-1-picrylhydrazyl (DPPH) Free Radical Scavenging Activity

The antioxidant activity was determined by the DPPH radical scavenging assay (DPPH test), which is based on the ability of DPPH, a stable free radical, DPPH solution will fade from purple in the presence of antioxidants. This test is a direct and reliable method for determining radical scavenging action [11]. Ascorbic acid was chosen as the reference antioxidant for this test. The freeze-dried bread and MF samples (1.0 g) were added to 3.0 mL of 80% ethanol and shaken at 150 rpm for 1 hour. The supernatant after the centrifugation for 10 min was used for experiment. Bread diet extract (0.2 mL) was added to 0.8 mL of 0.1 M Tris-HCl buffer (pH 7.4) and 1 mL of a methanol solution of 0.2 mM DPPH. Absorbance at 517 nm was determined after 30 min, and the antioxidant activity percentage was calculated by [(*A*_0_ − *A*_1_)/*A*_0_] × 100, where *A*_0_ is the absorbance of the control (used 80% ethanol in the stead of bread extract) and *A*_1_ is the absorbance of the extract. We also examined the antioxidant activity of white wheat flour, Steinmetz flour, whole-wheat flour, rye flour, and sesame by the same method (DPPH radical scavenging assay).

### 2.5. Serum Levels of 8-OHdG

8-Hydroxy-deoxyguanosine (8-OHdG) is a biomarker of oxidative DNA damage [12]. The levels of 8-OHdG in DNA and in the urine are known to be elevated in patients with malignancies [13]. We used serum levels of 8-OHdG as the biomarker of oxidative damage. Serum 8-OHdG levels were measured by the enzyme-linked immunosorbent assay (8-OHdG Chick high sensitivity; Japan Institute for the Control of Aging, Fukuroi, Japan).

### 2.6. Analysis of Aberrant Crypt

After animals were sacrificed, colons were quickly removed, flushed with saline solution, and longitudinally slit-opened from cecum to anus, placed on a paper towel, and fixed in 10% buffered formalin for 24 h. Next, the colons were stained with 0.2% methylene blue for 15–30 min and then placed on a glass slide with the luminal side up. Viewing the stained colons under a light microscope at a magnification of ×20–30, the presence of aberrant crypt foci (ACF) was assessed to determine the number of ACF and aberrant crypt (AC) and crypt multiplicity (AC/ACF) in DMH-treated rats.

### 2.7. Histological Analysis

After counting the ACF, the fixed colons were embedded in paraffin. Sections (4 *μ*m thick) of formalin-fixed, paraffin-embedded, colon tissues were prepared and stained with hematoxylin and eosin (HE) and examined under a light microscope (Olympus, Japan). Tumours were classified into two types: adenomas (grade of dysplasia: mild dysplasia, moderate dysplasia, and severe dysplasia) and adenocarcinomas (well differentiated tubular adenocarcinoma, moderately differentiated tubular adenocarcinoma, poorly differentiated adenocarcinoma, signet ring cell carcinoma, and mucinous carcinoma).

### 2.8. Statistical Analysis

The Statcel software package (KaleidaGraph Version 4.1) was used for analysis. The *α*2 test, Fisher's exact test, and *t*-test (Statcel, The Useful Addin Forms on Excel, 2nd ed.) were used for comparison of data between the two groups. A *P* value < 0.05 was considered statistically significant.

## 3. Results

### 3.1. Body Weight

Table 4 shows the body weight of the rats from the different groups at the end of the experiment. The final body weight of MF diet group rats was significantly higher compared to the rats in various bread diet groups; however, the final body weight in various bread groups was not significantly different. Food consumption was similar among these groups rats (Table 4).

### 3.2. The DPPH Free Radical Scavenging Rate

The DPPH free radical scavenging rates of the tested breads and MF diet are shown in Table 5. The Steinmetz Three-Grain bread and Steinmetz Country bread had higher antioxidant activity than the White bread and MF. Steinmetz Three-Grain bread showed the strongest antioxidant activity (64.1 ± 3.8%), Steinmetz Country bread showed moderate antioxidant activity (46.9 ± 1.9%), and White bread showed the weakest antioxidant activity among the three kinds of bread (7.2 ± 0.4%). The antioxidant activity of MF is (9.1 ± 0.7%).

The DPPH free radical scavenging rates of the tested raw materials of the breads are shown in Table 6. Steinmetz flour, Whole-wheat flour, rye flour, and sesame had higher antioxidant activity than white wheat flour. Sesame showed the strongest antioxidant activity (95.3 ± 1.7%), Steinmetz flour (75.3 ± 3.1%), whole-wheat flour (80.5 ± 1.9%), and rye flour (64.5 ± 3.8%) showed moderate antioxidant activity, and white wheat flour (36.9 ± 8.2%) showed the weakest antioxidant activity among the raw materials of the breads.

### 3.3. Serum Levels of 8-OHdG

The 8-OHdG serum levels in the four rat groups are shown in Table 7. The Steinmetz Three-Grain bread (A) group and the Steinmetz Country bread (B) group had a lower 8-OHdG level than that in the White bread (C) group and MF diet (D) group. The A group rats showed the lowest levels of 8-OHdG (0.19 ± 0.08%), while the C and D group rats showed the highest levels of 8-OHdG ((0.60 ± 0.09%) and (0.62 ± 0.09%)).

### 3.4. Colonic ACF

**Figure 2:**
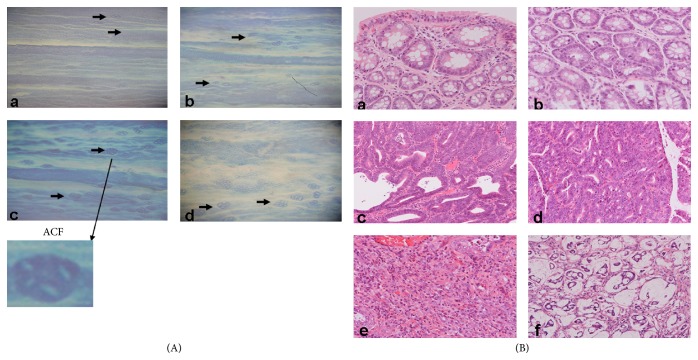
DMH-induced ACF and colorectal tumours in rats treated with DMH. (A) ACF expression in the colon of rats treated with DMH and stained with 0.5% methylene blue. (a) ACF expression in the colon from a rat of the Steinmetz Three-Grain bread group. Arrows point to two and three crypts in the colon. (b) ACF expression in the colon from a rat of the Steinmetz Country bread group. Arrows point to two and three crypts in the colon. (c) ACF expression in the colon from a rat of the White bread group. Arrows point to multiple crypts in the colon. (d) ACF expression in the colon from a rat of the MF diet group. Arrows point to multiple crypts in the colon. (B) DMH-induced colorectal tumours in rat. (a) Adenoma with mild dysplasia. (b) Adenoma with severe dysplasia. (c) Well differentiated tubular adenocarcinomas. (d) Moderately differentiated tubular adenocarcinomas. (e) Poorly differentiated adenocarcinomas. (f) Mucinous adenocarcinomas. Hematoxylin and eosin stains.

The effect of the tested breads on the growth and development of DMH-induced ACF in rats is shown in Figure 2(A) and Table 8. All rats treated with DMH showed a 100% incidence. However, group A showed a significantly lower average number of ACF, AC, and crypt multiplicity than group C or D did (Table 8). Crypt multiplicity was significantly lower in group B rats than in group C rats.

### 3.5. Colon Tumours

The histopathological findings are summarized in Table 9 and Figure 2(B). Colon epithelial lesions in rats were divided into adenomas and adenocarcinomas (Table 9). Rats from groups A and B had significantly fewer colon adenomas and adenocarcinomas than rats from groups C and group D did. The number of mild and moderate-grade dysplasia adenomas was significantly smaller in the Steinmetz Three-Grain bread group compared with that in the White bread and MF group (Table 9).

## 4. Discussion

Bread as a staple food product represents an important source of dietary fibre and various ingredients, which could influence chemoprevention of colon cancer. Our present study showed that rats from the Steinmetz Three-Grain bread group had the lowest incidence of ACF, adenomas, and adenocarcinomas in the colon and the strongest antioxidant effect, while rats from the White bread group had the highest incidence of ACF, adenomas, and adenocarcinomas in the colon and the weakest antioxidant effect among the three kinds of bread groups. These results suggest that the different ingredient combinations of the three breads have different functions. The content of fiber of 100 g Steinmetz Three-Grain bread, Steinmetz Country bread is high (8.2 g, 7.6 g) but of White bread is low (2.7 g) (Table 1). The ingredients of the breads are shown in Table 2(a). White bread was made from white wheat flour (100%) only; Steinmetz Country bread was made from Steinmetz flour (72.6%) and rye flour (27.4%); and Steinmetz Three-Grain bread was made from Steinmetz flour (75%), whole-wheat flour (15%), and rye flour (10%) and also sesame was added (Table 2(a)). We also found Steinmetz flour, rye flour, and whole-wheat flour and sesame contain a lot of vitamins, fibers, and minerals; however, white wheat flour contains small quantities of vitamins, fibers, and minerals (Table 2(b)). The Steinmetz Three-Grain bread and the Steinmetz Country bread have various antioxidant components.

Steinmetz flour was developed by the German Steinmetz Company. Grain only removes the hull; the aleurone layer, germ, and endosperm are preserved by the patented Steinmetz process. All vitamins and minerals are preserved because of the Steinmetz process (Table 2(b)) (Steinmetz; http://www.premium-flour.com). Our results have also shown that wheat flour prepared by the Steinmetz technology has high antioxidant capacity, commercialization by Andersen Japan. Steinmetz flour has higher antioxidant activity than white wheat flour does (Table 6). Rye grain and whole-wheat flour and sesame also contain a lot of vitamins and minerals (Table 2(b)). Rye grain contains a large variety of substances, including ones that are biologically active and demonstrate antioxidative properties, which include free radical scavengers, reducing agents, potential complexers of prooxidant metals, and quenchers of singlet oxygen formation [14]. It has been shown that rye bread showed better antioxidative properties and higher antioxidant contents when compared to wheat roll [15]. Another study has also shown that, compared with wheat bread, rye breads, which are an important source of B vitamins, showed better antioxidant properties. Therefore, rye breads should be more widely recommended for human nutrition [16]. Moreover, whole-wheat flour and sesame have also shown good antioxidative properties (Table 4) [8, 17]. Our results showed two kinds of Steinmetz bread had the higher antioxidant activity and suggest Steinmetz flour food may have a health benefit.

DMH is a procarcinogen that is metabolized to a methyl free radical, generating hydroxyl radicals or hydrogen peroxide in association with metal ions, which may contribute to the initiation of cancer and lipid peroxidation [10]. DMH treatment induced oxidative stress and early inflammatory and tumour promotion response in the colons of Wistar rats [10, 18]. Oxidative stress and inflammation play important roles and have been shown to influence tumour initiation and promotion [19]. In our results, the Steinmetz Three-Grain and Steinmetz Country bread groups also had a lower incidence of ACF adenomas and adenocarcinomas in the colon than the White bread group. Three-Grain bread and the Steinmetz Country bread may inhibit the formation and growth of tumours through antioxidation. Our results demonstrated that two kinds of Steinmetz bread had the higher antioxidant activity than White breads and MF, by the DPPH method (Table 5). Moreover, the Steinmetz Three-Grain bread (A) group and the Steinmetz Country bread (B) group had a lower 8-OHdG serum level than that in the White bread (C) and MF (D) group. 8-Hydroxy-deoxyguanosine (8-OHdG) is a biomarker of oxidative DNA damage [12]. The antioxidant activity of Steinmetz bread may reduce serum levels of 8-OHdG or in other words reduce the oxidative damage.

The abovementioned bread components not only have antioxidant functions, but also have anti-inflammation functions. Inflammation-related processes have also been shown to be involved in the development of both human and DMH-induced colon carcinogenesis [19]. COX-2, an important inflammatory marker, is, during inflammation, upregulated in adenomas and overexpressed in colon cancer [20, 21]. Increased expression of Cox-2 has also frequently been observed in DMH-induced colon adenocarcinomas, adenomas, and ACF with dysplasia [19]. The role of COX-2 in DMH/AOM-induced colon carcinogenesis and its involvement in different pathways of carcinogenesis is excellently described [22]. Wheat bran can inhibit the expression of Cox-2 in colon tissues induced by DMH injection in rats [23]. Steinmetz flour is that grain only removes the shell, but the Aleurone layer, germ, and endosperm are preserved by the patented STEINMETZ process, preserving more vitamins and minerals than wheat bran and having better anti-inflammatory effect than wheat bran. The Steinmetz bread may inhibit the formation and growth of tumours through anti-inflammation. Moreover, Rye flour and sesame also have other anti-tumour functions. Sesame and sesamin, a lignan of sesame, induce apoptosis and can mediate anti-inflammatory, antiproliferative, and antiangiogenic effects against cancer through the inhibition of NF-*κ*B and NF-*κ*B-regulated gene products, in a wide variety of human tumour cells [24, 25]. Whole grains and cereal fibre have anti-inflammatory properties [26]. Benzoxazinoids are also important phytochemicals found in wheat and rye that are associated with anticancer, antiallergy, and anti-inflammation activity [27].

Another study also showed rye bread consumption during adolescence may be associated with a reduced risk of prostate cancer [28]. The effect of rye whole grain may be related to the inhibition of prostate cancer progression caused by decreased exposure to insulin, as indicated by plasma insulin and urinary C-peptide excretion [29]. In addition, vitamins, minerals, and phytic acid in rye may provide protection against breast cancer [27]. These reports support our results that Steinmetz Three-Grain bread and Steinmetz Country bread can inhibit growth of tumours.

## 5. Conclusion

In summary, the current study suggests that, in contrast to White bread, Steinmetz Three-Grain bread and Steinmetz Country bread may inhibit colorectal cancer progression and may be useful for prevention of human colorectal cancer. Different ingredients of bread may relate to suppression of carcinogenesis, resulting from experiment of nutrimental imbalance like diet of only White bread.

## Figures and Tables

**Table 1:** Nutritional information of the breads.

100 g content	Bread		
	Steinmetz Three-Grain	Steinmetz Country	White
Energy (kcal)	256	241	244
Water (g)	41.9	39.8	40.5
Protein (g)	8.9	8.2	8.6
Lipid (g)	7.4	1.8	2.5
Dietary fiber (g)	8.2	7.6	2.7
Carbohydrate (g)	39.3	48.2	46.8
Ash (g)	2.5	2	1.5
Sodium (g)	445	463	470
Sodium chloride (g)	1.1	1.2	1.2

**(a):** 

100 g content	Bread		
	Steinmetz Three-Grain	Steinmetz Country	White
Steinmetz flour (g)	75	72.6	
White wheat flour (g)			100
Whole-wheat flour (g)	15		
Rye flour (g)	10	27.4	
Sesame (g)	20		
Sour kind dough (g)		6.2	
Salt (g)	2.5	1.9	1.9
Yeast (g)	2	0.9	2
Sugar (g)	1.4		2
Oil and fats (g)			2

**(b):** 

100 g content	Rye flour	Steinmetz flour	Whole-wheat flour	Sesame	White wheat flour
Calories (kcal)	359	366	357	564	349
Protein (g)	9	13.8	13	17.8	13
Total fat (g)	2.7	3.1	2.9	53.7	1.8
Carbohydrate (g)	74.7	70.8	69.6	19	70.3
Ash (g)	1.6	1.3	1.6	4.4	0.4
Dietary fiber (g)	12.9	5.6	12.1	12.6	2.7
K (mg)	140	214	355	410	80
Mg (mg)	30	88	150	360	23
Fe (mg)	1.5	2.3	3.9	9.9	1
VE (mg)	1	2.8	3.8	24.1	0.5
VB1 (mg)	0.15	0.35	0.33	0.49	0.10
Niacin (mg)	0.6	3.1	5.6	5.3	0.9

**Table 3:** Groups of experimental rats.

Group	Diet	Number of rats
A	DMH + Steinmetz Three-Grain bread	10
B	DMH + Steinmetz Country bread	10
C	DMH + White bread	11
D	DMH + MF (control)	11

**Table 4:** Average body weight of the rats at the end of the experiment and average food consumption.

Group	Body weight (g)	Food consumption of rats/day (g)
A	359.2 ± 16.8^a^	16.1 ± 2.5
B	325.9 ± 50.4^b^	16.8 ± 2.2
C	339.3 ± 20.9^b^	16.0 ± 2.7
D	392.7 ± 29.5	15.2 ± 2.3

^a^ *P* < 0.05 and ^b^ *P* < 0.01 versus D (MF) group.

**Table 5:** The 2,2-diphenyl-1-picrylhydrazyl (DPPH) free radical scavenging rate in the tested breads and MF diet.

Diet	DPPH free radical scavenging rate (%)
Steinmetz Three-Grain bread	64.1 ± 3.8^a,b^
Steinmetz Country bread	46.9 ± 1.9^a,b^
White bread	7.2 ± 0.4
MF	9.1 ± 0.7

0.2 mM DPPH free radical scavenging rate (%) of 0.1 g bread.

^a^ *P* < 0.01, versus White bread.

^b^ *P* < 0.01, versus MF.

**Table 6:** The 2,2-diphenyl-1-picrylhydrazyl (DPPH) free radical scavenging rate in the tested raw materials of the breads.

The raw materials of the breads	DPPH free radical scavenging rate (%)
White wheat flour	36.9 ± 8.2
Steinmetz flour	75.3 ± 3.1^∗∗^
Whole-wheat flour	80.5 ± 1.9^∗∗^
Rye flour	64.5 ± 3.8^∗∗^
Sesame	95.3 ± 1.7^∗∗^

^∗∗^ *P* < 0.01, versus white wheat flour.

**Table 7:** Serum levels of 8-hydroxy-deoxyguanosine (8-OHdG) in the three rat groups.

Group	Serum levels of 8-OHdG (ng/mL)
A	0.19 ± 0.08^a,b^
B	0.39 ± 0.08^a,b^
C	0.60 ± 0.09
D	0.62 ± 0.09

^a^ *P* < 0.01, versus C (White bread) group.

^b^ *P* < 0.01, versus D (MF) group.

**Table 8:** Aberrant crypt foci (ACF).

Group	ACF formation in rat colon		
	Number of ACF	Number of AC	Crypt multiplicity
			(AC/ACF)
A	159.3 ± 67.6^a^	546.7 ± 242.9^a,b^	3.4 ± 0.3^a^
B	188.8 ± 61.8	632.6 ± 257.6	3.3 ± 0.4^c^
C	237.4 ± 80.4	905.5 ± 323.4	3.8 ± 0.4
D	215.5 ± 62.4	800.1 ± 284.7	3.7 ± 0.4

^a^ *P* < 0.05 and ^c^ *P* < 0.05, versus C (White bread) group.

^b^ *P* < 0.05, versus D (MF) group.

AC: aberrant crypt.

**Table 9:** Number of colon adenomas and adenocarcinomas per rat.

Group	Number of colon adenomas per rat				Number of colon adenocarcinomas per rat
	Total	Mild-grade dysplasia	Moderate-grade dysplasia	Severe-grade dysplasia	
A	9.5 ± 4.81^a,b^	7.0 ± 3.71^a,d^	2.2 ± 1.75^a,b^	0.4 ± 0.52	0.50 ± 0.71^a^
B	19.1 ± 8.37^c^	11.5 ± 5.73	7.0 ± 4.14	0.6 ± 0.74	0.80 ± 1.03^c^
C	28.9 ± 10.1	16.6 ± 5.50	11.1 ± 5.22	1.2 ± 1.33	1.91 ± 1.14
D	23.5 ± 12.3	12.2 ± 6.52	10.4 ± 6.17	1.0 ± 1.18	1.18 ± 1.17

^a^ *P* < 0.01 and ^c^ *P* < 0.05, versus C (White bread) group.

^b^ *P* < 0.01, ^d^ *P* < 0.05, versus D (MF) group.

## References

[1] Garcia M., Jemal A., Ward E. M. (2007). *Global Cancer Facts and Figures*.

[2] MacDonald R. S., Wagner K. (2012). Influence of dietary phytochemicals and microbiota on colon cancer risk. *Journal of Agricultural and Food Chemistry*.

[3] Willett W. C., Stampfer M. J., Colditz G. A., Rosner B. A., Speizer F. E. (1990). Relation of meat, fat, and fiber intake to the risk of colon cancer in a prospective study among women. *The New England Journal of Medicine*.

[4] Sun Z., Liu L., Wang P. P. (2012). Association of total energy intake and macronutrient consumption with colorectal cancer risk: results from a large population-based case-control study in Newfoundland and Labrador and Ontario, Canada. *Nutrition Journal*.

[5] Otani T., Iwasaki M., Ishihara J., Sasazuki S., Inoue M., Tsugane S. (2006). Dietary fiber intake and subsequent risk of colorectal cancer: the Japan Public Health Center-based prospective study. *International Journal of Cancer*.

[6] McCullough M. L., Robertson A. S., Chao A. (2003). A prospective study of whole grains, fruits, vegetables and colon cancer risk. *Cancer Causes and Control*.

[7] Bingham S. A., Day N. E., Luben R. (2003). Dietary fibre in food and protection against colorectal cancer in the European Prospective Investigation into Cancer and Nutrition (EPIC): an observational study. *The Lancet*.

[8] Gil A., Ortega R. M., Maldonado J. (2011). Wholegrain cereals and bread: a duet of the Mediterranean diet for the prevention of chronic diseases. *Public Health Nutrition*.

[9] Baskar A. A., Ignacimuthu S., Paulraj G. M., Al Numair K. S. (2010). Chemopreventive potential of *β*-sitosterol in experimental colon cancer model-an *in vitro* and *in vivo* study. *BMC Complementary and Alternative Medicine*.

[10] Hamiza O. O., Rehman M. U., Tahir M. (2012). Amelioration of 1,2 dimethylhydrazine (DMH) induced colon oxidative stress, inflammation and tumor promotion response by tannic acid in wistar rats. *Asian Pacific Journal of Cancer Prevention*.

[11] Hasan S. M. R., Hossain M. M., Akter R., Jamila M., Mazumder M. E. H., Rahman S. (2009). DPPH free radical scavenging activity of some Bangladeshi medicinal plants. *Journal of Medicinal Plants Research*.

[12] Kasai H., Nishimura S. (1984). Hydroxylation of deoxyguanosine at the C-8 position by ascorbic acid and other reducing agents. *Nucleic Acids Research*.

[13] Tagesson C., Kallberg M., Klintenberg C., Starkhammar H. (1995). Determination of urinary 8-hydroxydeoxyguanosine by automated coupled-column high performance liquid chromatography: a powerful technique for assaying in vivo oxidative DNA damage in cancer patients. *European Journal of Cancer Part A: General Topics*.

[14] Ziclinski H. (2002). Low molecular weight antioxidants in the cercal grains. *Polish Journal of Food and Nutrition Sciences*.

[15] Michalska A., Ceglinska A., Amarowicz R., Piskula M. K., Szawara-Nowak D., Zielinski H. (2007). Antioxidant contents and antioxidative properties of traditional rye breads. *Journal of Agricultural and Food Chemistry*.

[16] Martinez-Villaluenga C., Michalska A., Frias J., Piskula M. K., Vidal-Valverde C., Zieliński H. (2009). Effect of flour extraction rate and baking on thiamine and riboflavin content and antioxidant capacity of traditional rye bread. *Journal of Food Science*.

[17] Wu W.-H., Kang Y.-P., Wang N.-H., Jou H.-J., Wang T.-A. (2006). Sesame ingestion affects sex hormones, antioxidant status, and blood lipids in postmenopausal women. *Journal of Nutrition*.

[18] Khan R., Sultana S. (2011). Farnesol attenuates 1,2-dimethylhydrazine induced oxidative stress, inflammation and apoptotic responses in the colon of Wistar rats. *Chemico-Biological Interactions*.

[19] Perše M., Cerar A. (2011). Morphological and molecular alterations in 1,2 dimethylhydrazine and azoxymethane induced colon carcinogenesis in rats. *Journal of Biomedicine and Biotechnology*.

[20] Eberhart C. E., Coffey R. J., Radhika A., Giardiello F. M., Ferrenbach S., Dubois R. N. (1994). Up-regulation of cyclooxygenase 2 gene expression in human colorectal adenomas and adenocarcinomas. *Gastroenterology*.

[21] Sano H., Kawahito Y., Wilder R. L. (1995). Expression of cyclooxygenase-1 and -2 in human colorectal cancer. *Cancer Research*.

[22] Takahashi M., Wakabayashi K. (2004). Gene mutations and altered gene expression in azoxymethane-induced colon carcinogenesis in rodents. *Cancer Science*.

[23] Kumar A., Singh N. K., Sinha P. R., Kumar R. (2010). Intervention of Acidophilus-casei dahi and Wheat bran against molecular alteration in colon carcinogenesis. *Molecular Biology Reports*.

[24] Harikumar K. B., Sung B., Tharakan S. T. (2010). Sesamin manifests chemopreventive effects through the suppression of NF-*κ*B-regulated cell survival, proliferation, invasion, and angiogenic gene products. *Molecular Cancer Research*.

[25] Fujimoto A., Shingai Y., Oyama T. B. (2010). Apoptosis-inducing action of two products from oxidation of sesamol, an antioxidative constituent of sesame oil: a possible cytotoxicity of oxidized antioxidant. *Toxicology in Vitro*.

[26] Bickers D. R., Athar M. (2006). Oxidative stress in the pathogenesis of skin disease. *Journal of Investigative Dermatology*.

[27] Tanwir F., Fredholm M., Gregersen P. L., Fomsgaard I. S. (2013). Comparison of the levels of bioactive benzoxazinoids in different wheat and rye fractions and the transformation of these compounds in homemade foods. *Food Chemistry*.

[28] Torfadottir J. E., Valdimarsdottir U. A., Mucci L. (2012). Rye bread consumption in early life and reduced risk of advanced prostate cancer. *Cancer Causes and Control*.

[29] Landberg R., Andersson S.-O., Zhang J.-X. (2010). Rye whole grain and bran intake compared with refined wheat decreases urinary C-peptide, plasma insulin, and prostate specific antigen in men with prostate cancer. *Journal of Nutrition*.

